# Water jet as a novel technique for enamel drilling *ex vivo*

**DOI:** 10.1371/journal.pone.0254787

**Published:** 2021-07-23

**Authors:** Chang Liu, Rourong Chen, ChengZao Han, Xiaoqin Pi, Shuli Chang, Han Jiang, Xinping Long, Minquan Du

**Affiliations:** 1 School & Hospital of Stomatology, The State Key Laboratory Breeding Base of Basic Science of Stomatology (Hubei-MOST) & Key Laboratory of Oral Biomedicine Ministry of Education, Wuhan University, Wuhan, Peoples R China; 2 Wuhan Univ, Sch Power & Mech Engn, State Key Lab Water Resources & Hydropower Engn S, Wuhan, Peoples R China; University of Vigo, SPAIN

## Abstract

To investigate the usage of a water jet for enamel drilling *ex vivo*, 210 individual extracted molars without lesions or fillings were collected. Then, the specimens were drilled by a water jet or a high-speed dental drill. The cavities of 50 teeth were reconstructed digitally by micro-computed tomography (micro-CT) to measure the height and width. The cavities of 10 teeth were longitudinally incised and their surfaces were observed by scanning electronic microscopy (SEM). After the cavities were filled, 50 fillings were vertically incised. The bonding interface between tooth and filling was observed by SEM. 50 teeth with fillings were stained in 0.1% rhodamine B solution, and then the dye penetration between tooth and filling was observed under the stereomicroscope and confocal laser scanning microscopy (CLSM). The bonding strength between enamel and filling of 50 teeth was simulated and predicted with finite element analysis (FEA). At 140–150 MPa and for 2–3 s, cavities were made with a depth of approximately 764 μm in each tooth. SEM showed the cavity surface in the water jet group had a more irregular concave and convex structure than that in the high-speed dental drill group. There was a trend that the microleakage and bonding width was smaller in the water jet group than in the high-speed dental drill group. FEA indicated that the stress on the resin surface was greater than on the enamel surface in the water jet group. Compared with the tooth drilled by a high-speed dental drill, the tooth drilled by a water jet gained better retention of the filling material and suffered less bonding strength on the enamel surface. Water jet drilling is effective for enamel drilling.

## Introduction

The traditional enamel drilling technique is the high-speed dental drill. However, there are several shortcomings. The drilled tooth debris is not easily removed in time, so the surface of the cavity forms a smear layer [1], affecting the bonding effect of the filling [2]. Due to high speed, it is easy to cause iatrogenic soft tissue injury [3], even swallowing and aspiration by mistake. Heat, which is generated [4] during drilling without cooling, increases the risk of irreversible pulpitis [5]. Moreover, heat induces the body to synthesize proteins that can mediate the immune response of cells [6]. Thermal stimulation can up-regulate TNF-α expression through TRPV1 activation in human periodontal ligament cells [7]. Noises cause dental fear and hearing impairment in patients, especially children, which is not conducive to patients’ cooperation [8, 9]. After the operation, the water ejected from the drilling process can be sucked back into the water-supply tubing, which can easily cause iatrogenic infection [10]. Additionally, a high-speed dental drill is required to be regularly lubricated and maintained. Its structure is more complex and less reliable than other configurations, because of its rotating parts. Moreover, a high-speed dental drill is susceptible to wear and damage its engine, so it needs to be replaced frequently.

To avoid these shortcomings, researchers invented the water laser [11]. Water is energized by the laser with a specific wavelength and sprayed outwards at high speed. With this working principle, a water laser can drill teeth. During the process, water mist droplets are dispersed to protect normal tissues by removing heat and debris from the damaged tissue. However, water laser equipment is expensive. The optical fiber laser head is disposable and cannot be maintained. At present, the replacement is prohibitively expensive. In addition, studies have shown that water laser can still increase the temperature of the dental surface, pulp chamber, and soft tissue, though the water cooling significantly suppresses this effect compared to the case of the high-speed dental drill [12, 13]. These shortcomings may limit the application of water lasers in health care.

Therefore, it is of great significance to research and develop a new economic dental hard tissue drilling technology. As a high-energy cutting technology [14], the water jet drilling technique utilizes water with high pressure through nozzles of specific shape and size [15]. The water jet has been widely applied for cutting mineral stones, glass, and so on [16, 17]. In recent years, the water jet has been developed into a revolutionary drilling tool for soft tissues and hard tissues in surgery [18–22] and integrated into robotic surgery systems [23–25].

When the water jet is used, its thermal deformation effect can be neglected because the heat generated by kinetic energy is rapidly removed by water. In addition, the main working medium is water, which has a wide range of sources. Compared with the traditional high-speed dental drill and laser technique, the working end of the water jet is a simple structure without moving parts. Therefore, the high-pressure water jet drilling technique has some advantages, such as high efficiency, environmental protection, as well as energy and cost savings [26, 27]. Moreover, the high-pressure water jet drilling technique is good for our health. Although few relevant studies have been reported, the advantages of the high-pressure water jet technology and its successful application in the medical field make it a potential application prospect in dental hard tissue drilling. With this case, we hypothesized that the water jet drilling would be effective for enamel drilling (null hypothesis: the water jet drilling technique with suitable parameters can’t be used to drill enamel effectively).

To explore this hypothesis, we performed this study to work out whether a high-pressure water jet drilling technique can be introduced into enamel drilling. At the same time, we carried out basic research on the relevant mechanism including observation and measurement of the bonding interface, as well as prediction of the bonding strength.

## Materials and methods

### A. Teeth collection

The Ethics Committee of School & Hospital of Stomatology, Wuhan University in China reviewed and approved the present study in written form [2020B13]. All the molars had been removed during routine care, and no single tooth has been extracted to serve for our study. The study was carried out without human subjects, the Ethics Committee approved that molars were used for research without the donors consented in written form. None of the donors were from a vulnerable population.

Individual molars extracted in the clinic from June to December 2018 were selected without information that could identify individual participants. The inclusion criteria were as follows: 1. complete tooth, no cavity; 2. no crack on the tooth surface; 3. not discolored or bleached; and 4. no filling, veneer, or other restorations. Exclusion criteria: enamel hypoplasia, dental fluorosis, tetracycline teeth, and other hard tissue diseases. The specimens were screened using a stereomicroscope (Olympus SZ61-SET; Olympus, Tokyo, Japan) at 4 times magnification. The specimens were washed ultrasonically (KQ2200E; Kunshan Shumei, Kunshan, Jiangsu, China), rinsed with distilled water (UPW-1; Wuhan Jibairui, Wuhan, Hubei, China), soaked in 0.9% sodium chloride injection (H51021157; Sichuan Kelun, Sichuan, China) and stored at 4°C.

### B. Specimen preparation

**Fig 1:**
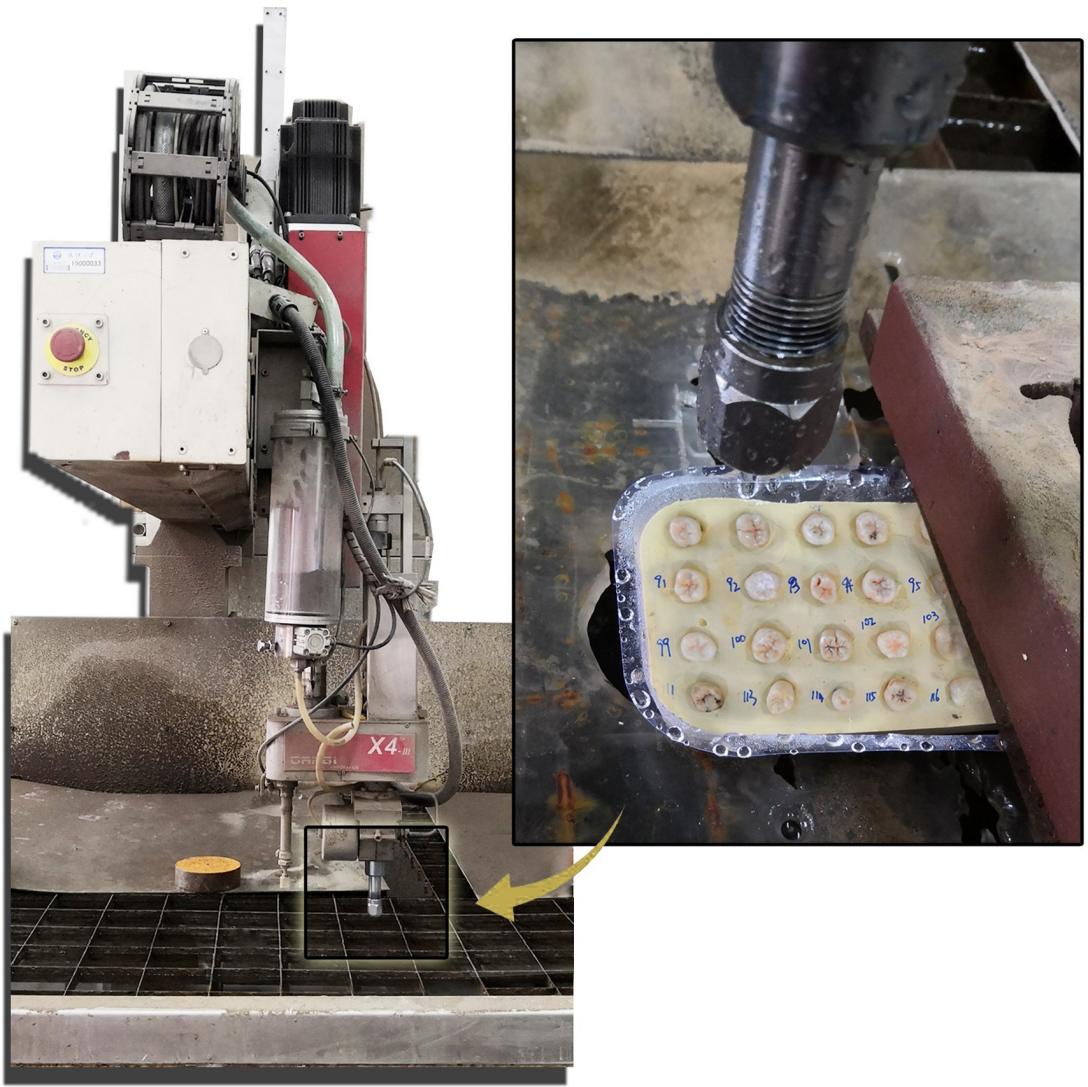
Specimen preparation.

The roots of teeth were all embedded in the anhydrite (PYD, Foshan, Guangdong, China) to ensure a stable position under the impact of the water jet (Fig 1). To reduce the energy loss of the water from the jet to the tooth surface, the target distance was set to 1 mm using a diameter nozzle of 1 mm. The depth and width of cavities were recorded. If the teeth appeared to have cracks or even fractures, the pressure and time were decreased. Otherwise, if the tooth surface was not drilled, the pressure and time were increased until the best variables were explored. The specimen preparations were done by one researcher.

### C. Specimen grouping

**Fig 2:**
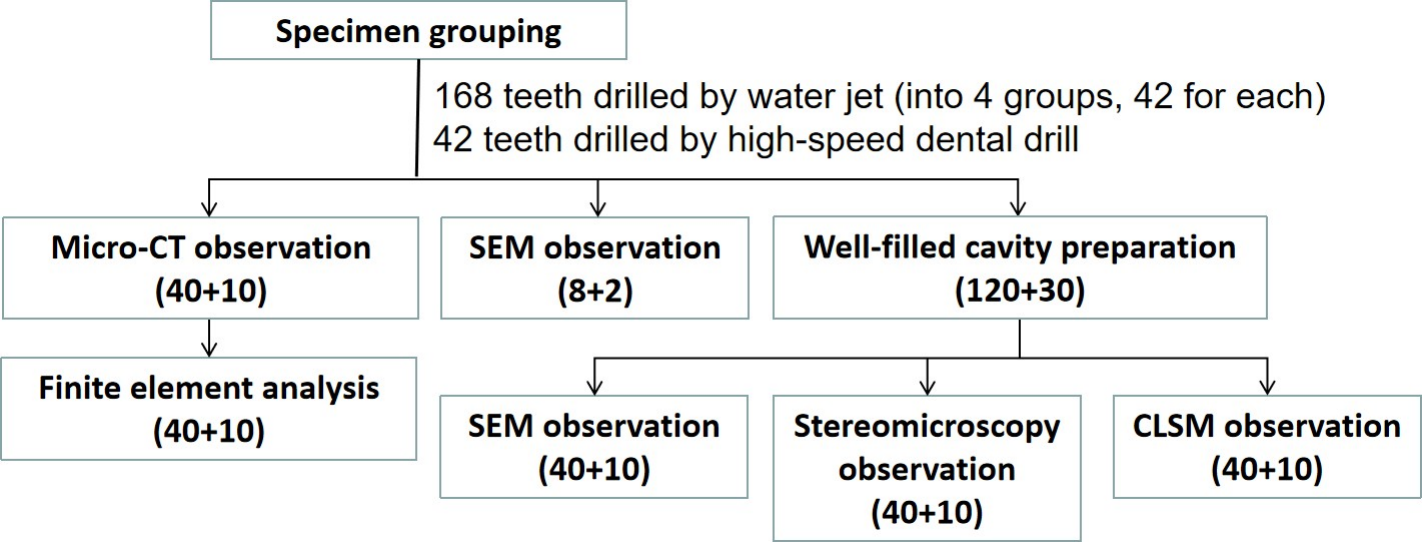
Flow diagram of specimen grouping.

According to different pressures and times from the above results, 168 teeth, including 4 groups (Fig 2), were randomly drilled by a digital control cantilever water jet cutting machine (DWJ1313-FC; Dadi, Jiangsu, Nanjing, China) as experimental groups. Then, 42 teeth were drilled by the high-speed dental drill (NSK, Tokyo, Japan) with fissure bur (FG700; Dentsply, Charlotte, NC, USA) to a depth of 1 mm as the control group. In other words, the selected teeth were grouped as follows: (1) For cavity observation and measurement, 50 teeth were observed by micro-computed tomography (micro-CT); the cavity surface of 10 teeth was observed by scanning electron microscopy (SEM). (2) For bonding interface observation and measurement, 50 teeth were measured for the width of the micro gap under SEM; 50 teeth were measured for the depth of microleakage under a stereo microscope, and then they were further measured for the depth of microleakage under confocal laser scanning microscopy (CLSM). (3) For the prediction of bonding strength, 3D models were analyzed by a series of computer software.

### D. Cavities observation and measurement by micro-CT

High-resolution micro-CT (SkyScan 1176; Bruker microCT, Kontich, Belgium) was used to observe the cavity. The data was reconstructed in NRecon (Micro Photonics, Allentown, PA, USA) and visualized in 3D models in CTvox (Blue Scientific, Cambridge, Cambridgeshire, UK), and the surface and height were measured by CTan (Blue Scientific, Cambridge, Cambridgeshire, UK). The parameters were as follows: The ray filter was Cu 0.1; the image resolution was 1024 × 1024 pixels; the voxel dimension was 9.00 μm, with values of reconstruction ranging from 0.01 to 0.04; and the gray values of measurement ranging from 50 to 255. Then, the data was modeled in Mimics Research 20.0 (Materialise, Plymouth, MI, USA) with the threshold set from 10 to max and smooth 30 to gain a part file. The data was remeshed in 3-Matic Research 12.0 (Materialise, Plymouth, MI, USA) to gain a model file. The buccolingual direction was set as the X-axis, mesiodistal direction as the Y-axis, and upwards orientation as the Z-axis. This procedure was done by one researcher blinded to the groups.

### E. Cavities surface observation by SEM

To gain a morphology image of the cavity surface, the cavities were incised longitudinally along the center of the cavities by a low-speed diamond slice disc (Low-Speed Precision Cutter IsoMet 11-1280-170; Buehler, IL, USA) under the condition of water cooling, following by cleaning with 0.9% sodium chloride injection and ultrasonically washing for 30 min. Then, the cavities were dried by vacuum-pumping equipment (Vacucell, München, Bavaria, Germany). The specimens adhered to a metal specimen loading platform, and then a metal film was deposited in a vacuum evaporator. These specimens were observed under SEM (LMH, MIRA 3; Teacan, Brno, Kohoutovice, Czech). The morphology of the cavity surface was examined, including the accumulation of enamel debris and the structure of the enamel rods and dentinal tubules. This procedure was done by one researcher blinded to the groups.

### F. Well-filled cavities preparations

The cavities were cleaned with 0.9% sodium chloride injection and filled with light-curing composite resins (high fluidity, Gradia Direct Flo; GC, Tokyo, Japan). The steps for filling according to the instructions were as follows: washing 20 s after acid etching (GLUMA Etch 35 Gel; Kulzer, Tokyo, Japan) for 30 s, gently blowing for 20 s, evenly applying adhesive with a small brush (M6500XS; TPC, CA, USA), blowing for 5 s, exposing to curing light (FD-L029A; Dentsply, Charlotte, NC, USA) for 20 s normally filling with light-curing composite resins and exposing to curing light again for 20 s. Then, the samples were cleaned with 0.9% sodium chloride injection again and ultrasonically washed for 30 min. After filling the cavity, the teeth were placed in a tube filled with 0.9% sodium chloride injection, and then the tube was immersed in a constant-temperature water bath (115VAC, SC 100 Digital Immersion Circulator, 100°C w/clamp; Thermo Fisher Scientific, Waltham, MA, USA) at 37°C for 24 h. One thousand cycles of temperature shifts were performed with a cold water bath 5 (±2)°C for 30 s and a hot water bath 55 (±2)°C for 30 s, of which the conversion time was 10 s. To avoid operator bias, the steps were performed by an experienced dentist blinded to the groups.

### G. Measurement of micro gaps at the bonding interface by SEM

After the cavities were filled, a low-speed diamond sand sheet was drilled vertically into the fillings’ surface under water cooling to expose the bonding interface. Surface treatment was carried out on the bonding interface side. First, 600, 800, 1200, and 2000 water-compatible sandpaper (Hengyu, Zhejiang, China) were gradually used to polish the surface in turn, and then the teeth were placed on a glass slide and dried by vacuum-pumping equipment.

Subsequently, the tooth surfaces were treated by fixation and drying. The specimens adhered to a metal specimen stage with an adhesive, and then a metal film was deposited in a vacuum evaporator. The micromorphological characteristics of the bonding interface between tooth and filling were observed under SEM. ImageJ software (image analyzing software, https://imagej.nih.gov/ij/) was used to measure the width of the micro gap between the light-cured composite resin filling and teeth. Six data points were measured for each axial wall (at 1/3, 2/3, and 3/3 of each side of the axial wall), and one data point was measured for the pulpal wall (at 1/2 of the axial wall). It was measured in triplicate to ensure the consistency of the experimental conditions, and therefore at least 21 data points were collected. The mean value was corresponding to the width of the micro gap. To avoid examiner bias, the specimens were measured by one examiner blinded to the groups.

### H. Observation of microleakage at the bonding interface by stereomicroscopy and CLSM

After cavities were filled, the teeth were washed and dried. Two layers of nail polish evenly coated all areas except the fillings, and the root apex was sealed with wax. Then, the specimens were placed in 0.1% rhodamine B (71036314, Hushi; Guoyao, Shanghai, China) solution and immersed at 37°C for 24 h. The excess dyes on the surface were removed with a soft brush under running water, and these teeth were stored in 4°C distilled water and kept in a dark place until incision. No loosening of the filling was found. Under the condition of water cooling, a low-speed diamond slice disc was used to incise the teeth longitudinally along the long axis of the filling. The dye penetration on the wall of the filling was observed and photographed under a stereomicroscope at 4 times magnification. To avoid examiner bias, the specimens were measured by one examiner blinded to the groups.

The dye penetration score was used for the evaluation as follows [28]:

Grade 0: Dye-free infiltration

Grade 1: Dye penetration not exceeding 1/3 of the hole depth

Grade 2: Dye penetration beyond 1/3 of the hole depth but not exceeding 2/3

Grade 3: Dye penetration beyond 2/3 of the hole depth but not reaching the pulpal wall

Grade 4: Dye penetration into the pulpal wall

To ensure the consistency of the experimental conditions, the remaining half of the filling was observed and photographed under CLSM (Fluoview FV1200; Olympus, Tokyo, Japan) with a magnification of 4 times at 589 nm. The edge of the lower extent of rhodamine B was observed.

### I. Prediction of bonding strength based on Finite Element Analysis (FEA)

After the cavities were filled, a model file for each cavity was obtained by micro-CT, and 3D-FEM models of idealized Class I filling with minimum clearance space were constructed by 3-Matic Research 12.0. Then, we predicted the bonding strength simulated with FEA by Abaqus 6.14 (Simulia, RI, USA). The behavior of small, symmetric cavities was studied using the finite element model [29].

Taking elasticity into account, the stresses and the displacements were calculated with their interaction. The model used associated material properties with the following parameters: Enamel and resin were modeled as isotropic. Young’s modulus of enamel was set to 48 MPa, while that of the resin was set to 12.5 MPa. Poisson’s ratio was assumed to 0.23 for enamel and 0.3 for resin [30]. The specimens were measured by one examiner blinded to the groups.

### J. Statistical analysis

The data was analyzed by SPSS 21.0 statistical software (SPSS, Chicago, IL, USA), and the measurement data are expressed as the mean (±SD). The Mann-Whitney U test was used to compare the difference between two independent samples. One-way ANOVA or the Kruskal-Wallis test compared the means of three or more independent groups. The difference was significant if p < 0.05.

## Results

### A. The 140 MPa 2 s water jet group drilled the deepest into the teeth cavity

**Fig 3:**
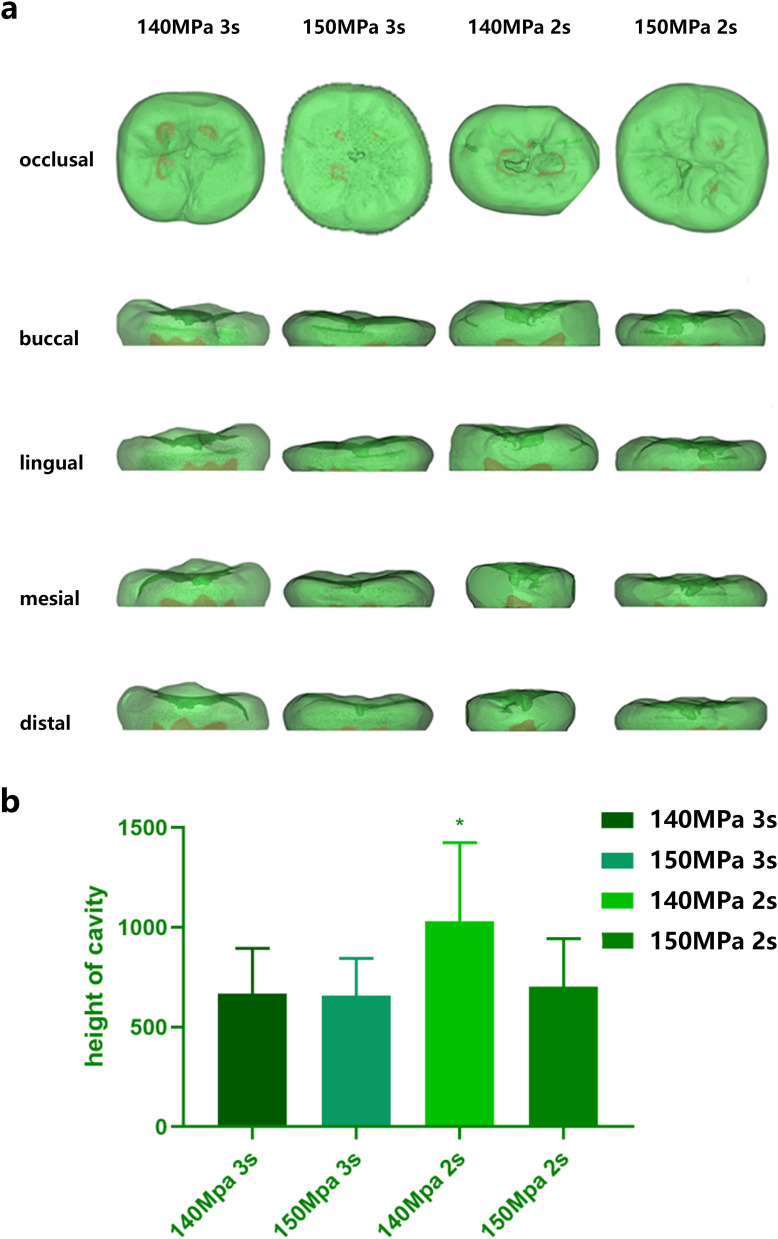
a, The 3D model of cavity drilling by a water jet. The cavities (including the innate pit and fissure) were shown in dark green. The pulp cavities were shown in light red. b, The height of the cavity prepared with a water jet or a high-speed dental drill (n = 10). The asterisk represents statistical significance among the 140 MPa, 2 s water jet group and other groups (p < 0.05). Data represent the mean (±SD).

There was no significant difference in the hardness among groups (p = 0.389). Fig 3A showed five views of representative reconstructed models in different groups. We recorded the success rate of drilling cavities as their depth over 100 μm. There was no significant difference in the success rate of drilling and surface area (p = 0.373) among groups, while a significant difference appeared in the depth of the cavity (p = 0.045, Table 1). In Tukey’s multiple comparisons test, we found statistical significance among the 140 MPa, 2 s water jet group and other groups (Fig 3B).

**Table 1:** Basic information about cavity drill with different pressure and time. Data represent the mean (±SD).

Group	Success rate of drilling (%)	Surface area (μm^2^)	Height (μm)
**140 MPa 3 s**	73.53	63066.13 (±25917.19)	667.19 (±227.29)
**150 MPa 3 s**	69.57	77903.98 (±31959.85)	658.18 (±185.76)
**140 MPa 2 s**	71.19	97992.63 (±39804.96)	1031.85 (±391.90)
**150 MPa 2 s**	83.87	75786.30 (±37878.94)	701.40 (±241.24)

### B. The water jet group produced a rough cavity wall and bottom

**Fig 4:**
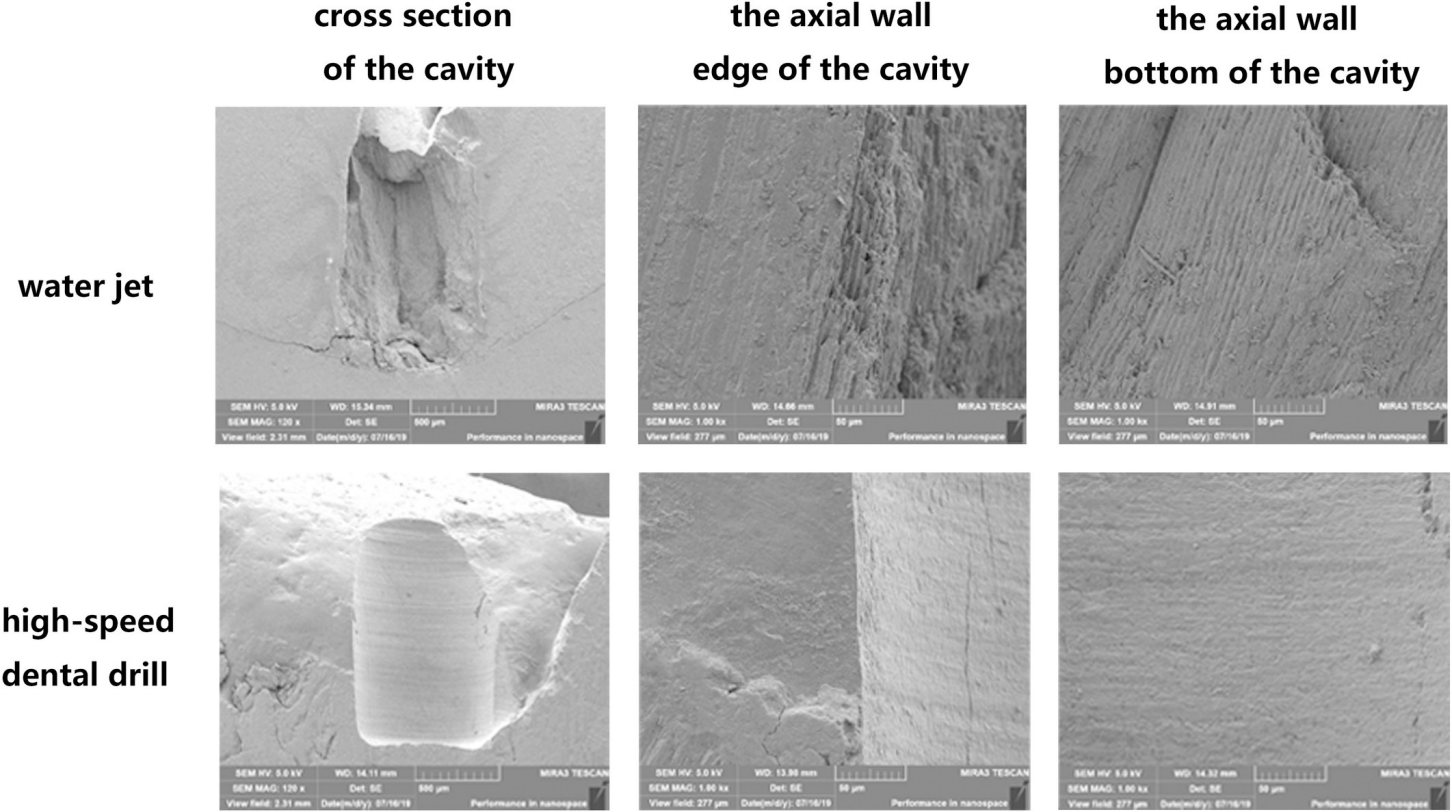
Observation of the drilling surface by SEM. SEM images of the cavities prepared with a water jet and a high-speed dental drill and their axial wall edge and the axial wall bottom.

The SEM images showed that the wall and bottom of the cavity prepared with a water jet were multiform, irregular, rough, and scaly with clear slender enamel pillars. There was a small amount of debris without signs of carbonization or damage from thermal melting. In the high-speed dental drill group, the surface was flatted and coated with a dense smear layer (Fig 4).

### C. The water jet group exhibited a slightly thinner interface width than the high-speed dental drill group

**Fig 5:**
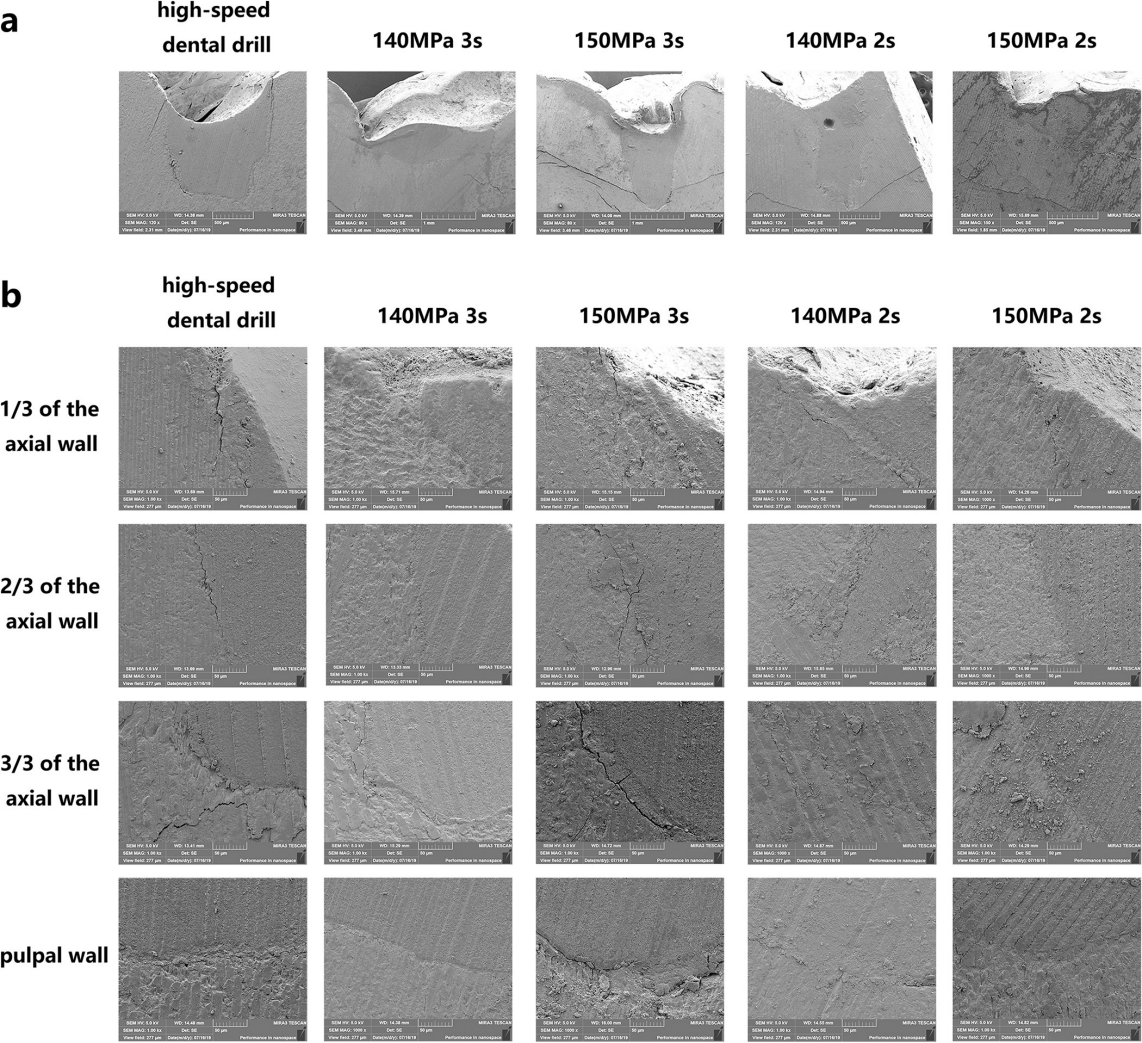
Observation of the bonding interface. a, SEM images of well-filled cavities prepared with a water jet or a high-speed dental drill. b, SEM images of the axis wall edge of well-filled cavities prepared with a water jet or a high-speed dental drill.

Under SEM, the interface of the water jet group was not as obvious as that of the high-speed dental drill group (Fig 5A). The width of the interface between tooth and filling formed by the water jet was slightly thinner than that formed by the high-speed dental drill without significant difference (Table 2), while there was a significant difference in the width of the interface at 1/3 of the axial wall (Fig 5B).

**Table 2:** The width of the interface between tooth and filling formed by a water jet preparation and a high-speed dental drill preparation. Data represent the mean (±SD).

Group (n = 10)		Length				
		Average	A	B	C	D
**High-speed dental drill**		1.22 (±1.01)	1.19 (±1.21)	0.71 (±0.27)	1.47 (±2.15)	1.54 (±1.94)
**Water jet**	**140 MPa 3 s**	0.65 (±0.16)	0.53 (±0.07)	0.58 (±0.11)	0.94 (±0.70)	0.61 (±0.27)
	**150 MPa 3 s**	0.70 (±0.18)	0.70 (±0.27)	0.64 (±0.15)	0.74 (±0.28)	0.76 (±0.24)
	**140 MPa 2 s**	0.93 (±0.39)	0.84 (±0.41)	1.09 (±0.69)	0.82 (±0.26)	1.01 (±0.55)
	**150 MPa 2 s**	0.75 (±0.15)	0.73 (±0.17)	0.63 (±0.19)	0.79 (±0.15)	1.09 (±0.52)
***p* value**^**a**^		0.089	0.166	0.016	0.507	0.245

A,B,C,D represent 1/3, 2/3, 3/3 of the axial wall, pulpal wall respectively.

^a^ *p* values were calculated using the one-way ANOVA.

### D. The water jet group showed better dye penetration scores than the high-speed dental drill group

**Fig 6:**
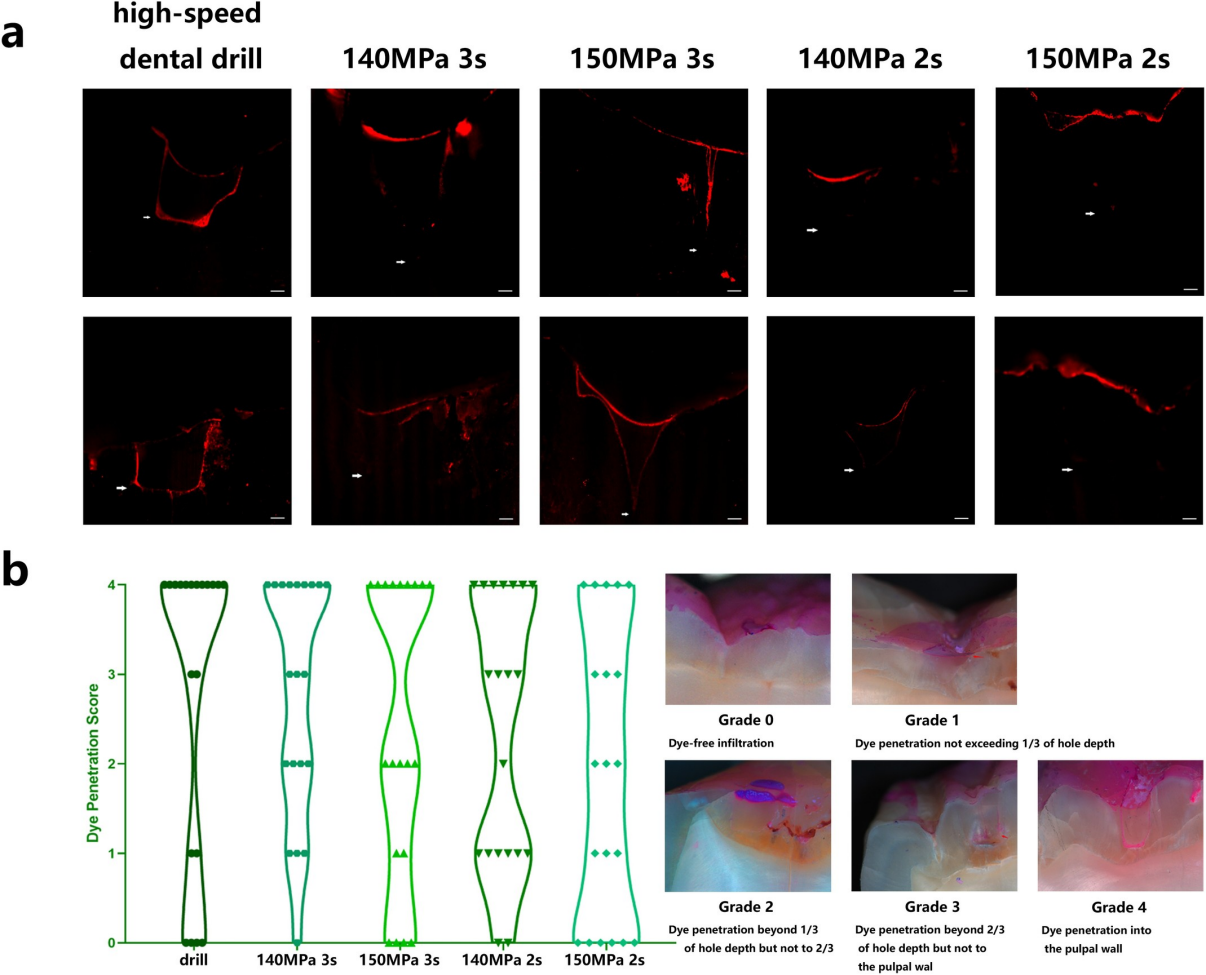
Observation of microleakage. a, CLSM images of microleakage of well-filled cavities prepared with a water jet or a high-speed dental drill (arrows show the bottom of the resin, scale bar = 200 μm). b, The dye penetration score under stereomicroscope observation of well-filled cavities prepared with a water jet or a high-speed dental drill in a violin plot (n = 20). The dye penetration score was used for the evaluation of example images (arrows show the bottom of dye penetration).

Under stereomicroscopy and CLSM, most microleakage was only existed on the wall and did not involve the bottom in the water jet group, while most microleakage was over the wall and bottom in the high-speed dental drill group (Fig 6A). Although there was no significant difference (p = 0.347) in the dye penetration scores among the water jet and the high-speed dental drill groups, a violin plot (Fig 6B) showed that it was easier to obtain a high score (penetration beyond 2/3 of cavity depth and even into the pulpal wall) when prepared with the high-speed dental drill.

### E. Water jet group might suffer from a lower tensile strength

**Fig 7:**
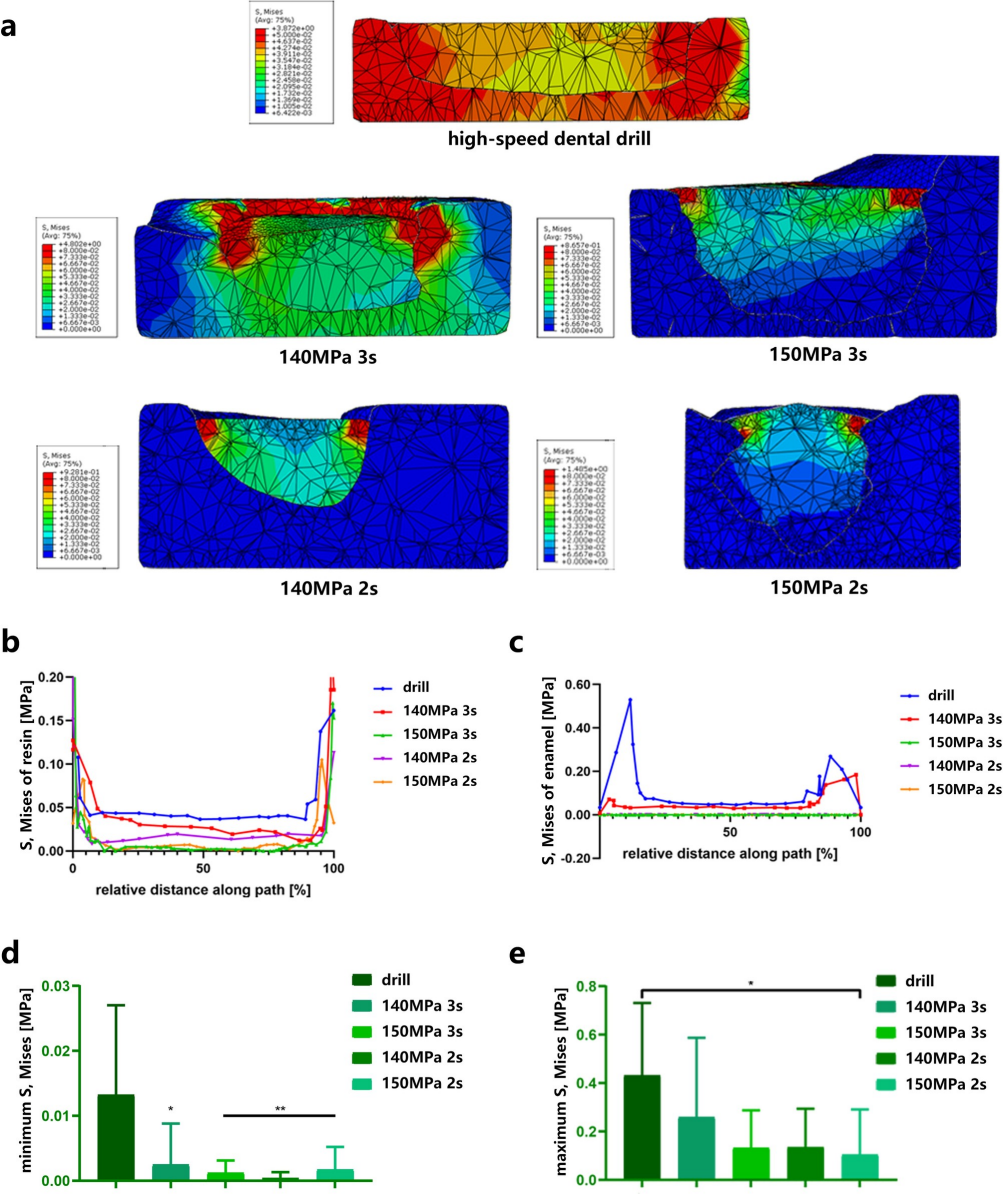
Prediction of bonding strength through simulation with FEA. **a**, Heat map of the Von Mises stress of the model based on the cavity prepared with a water jet or a high-speed dental drill. **b**, The Von Mises stress of the resin interface prepared with a water jet or a high-speed dental drill (n = 10). **c**, The Von Mises stress of the enamel interface prepared with a water jet or a high-speed dental drill (n = 10). **d**, The minimum Von Mises stress of the enamel interface prepared with a water jet or a high-speed dental drill (n = 10). The asterisk represents statistical significance between the high-speed dental drill group and the water jet group. Data represent the mean (±SD), *p < 0.05, **p < 0.01. **e**, The maximum Von Mises stress of the enamel interface prepared with a water jet or a high-speed dental drill (n = 10). The asterisk represents statistical significance between the high-speed dental drill group and the 150 MPa, 2 s water jet group, *p < 0.05. Data represent the mean (±SD).

With FEA, we focused on the tensile strength to assess the mechanical loading on teeth and filling. As shown in the heat map of Von Mises stress, with a water jet drilling, the stress predominated at the resin rather than at the enamel (Fig 7A). There was a tendency that, when drilled by a water jet, the bonding interface gained less Von Mises stress regardless of resin or enamel (Fig 7B and 7C). Although there was no significant difference in the Von Mises stress of resin (p = 0.502 for minimum Von Mises stress, p = 0.207 for maximum Von Mises stress), there was significantly less minimum Von Mises stress of enamel interface when drilled by a water jet (p = 0.001, Fig 7D). Regarding the maximum Von Mises stress, the teeth drilled by a water jet, especially with 150 MPa and 2 s, had lower maximum Von Mises stress at the teeth interface than at the enamel interface (p = 0.019, Fig 7E).

## Discussion

Although there had been no reports about the application of the water jet on the drilling of teeth, high-pressure water jet technology has made great progress in drilling materials with similar mechanical properties [23–25]. In this study, we found that teeth drilled by a water jet gained better retention of the filling material and suffered less bonding strength on the enamel surface. It is well demonstrated that the water jet drilling is effective for enamel drilling, providing an experimental basis for the promotion and development of clinical applications.

We had found through the pilot study that the water jet drilling only worked when it was within a certain range of pressure and time. The stress was concentrated on a region surface, causing the existence of a low-stress area and resulting in a stress shielding effect, so brittle failure appeared [31]. However, it is pointed out that spallation damage would occur only when the tensile stress was greater than the spallation strength (i.e., 0.5 tensile strength) of the hard brittle material, and the stress history should be taken into consideration [32]. Concerning time, extended time would increase the risk of dentin microcracks or even tooth fractures. However, if the time is too short, the pressure cannot reach the tooth surface. Therefore, in the application, suitable parameters including pressure and working time should be considered.

Tooth damage and micro cracks should be avoided. The study found that below 140 MPa, cavities could not be created; between 140 and 150 MPa for 2–3 s, it could drill a cavity in a tooth; and above 150 MPa, microcracks could be formed. Therefore, these specimens were divided into 4 groups: 140 MPa, 2 s; 140 MPa, 3 s; 150 MPa, 2 s and 150 MPa, 3 s. Nevertheless, the water jet pressure and distance are also the most important factors [33]. Hence, further research is needed.

While observing and measuring cavities, it was interesting that with increasing pressure or time, we gained cavities with smaller heights. The height of the cavity exhibited no time-pressure dependence over the effective and safe range. It even decreased while increasing the water jet pressure, which meant that the surface compressive residual stress could be decreased as a result of increasing the water jet pressure [34, 35]. Furthermore, in the initial contact of the water jet and enamel, the interaction process was idealized as the impact of a water column and a hard brittle material. Because of the high-speed water, its failure may result from the erosion damage of droplets, which act as small solid particles when striking high-speed aerospace equipment. When water sprayed into our innate pit and fissure, it formed a water cushion on the bottom of the cavity, which increased the resistance effect of the jet [36]. The cavities were more likely to become wider rather than deeper with higher pressure or longer drilling. Drilling with a water jet, we gained cavities shaped like inborn fissures, which can avoid the removal of excessive tooth tissue.

Drilling surface structure influenced the retention of the filling material [37, 38]. The results of surface structure and filling effect showed that the water jet was good for marginal adaptation efficiency and filling retention to a certain extent. The SEM results of this experiment showed that compared with the traditional drilling method, the water jet drilled enamel without a smudging layer and obvious thermal damage. The smear layer on the teeth caused by the traditional cavity preparation was an unfavorable factor affecting the retention of the filling and the shear strength of the teeth [39]. A smaller smear layer signified less need for acid etching and less damage. Furthermore, the specimen drilled by a water jet was characterized by a rough surface [40]. Formation of striation and exposure of enamel rods increased the area of adhesive bonding and the retention of filling [2].

Considering that the hybrid layer is also highly related to aging phenomena [41], we observed and measured its bonding interface, especially the microleakage and artificial aging effect. The results showed that the adhesion between tooth and filling indeed directly affected the long-term effect of filling. Although there was no significant difference in the width of the interface generally, there was a significant difference at the top 1/3 of the axial wall. This difference might also be caused by the small expansion mentioned before, which can avoid bacteria leakage. Therefore, regarding dye penetration, a difference was observed. After the water jet drilled the teeth tissue, the area of microleakage was smaller, though there was no significant difference, and longer artificial aging might be needed.

To further verify the bonding strength, we predicted the bonding strength simulated with FEA. Surprisingly, the FEA results exhibited the same tendency as the microleakage results. When drilled by a water jet, the stress was more focused on resin filling than enamel. This might be because of the reflected secondary water jet. The axial wall of the cavity was not vertical to the pulpal wall [42, 43] and featured beveled edges. The bevel was so small that it could not be made by traditional cavity preparation. With this unique strength, such ingenious shapes dispersed the stress on the enamel and focus the stress on the resin. Nevertheless, it must be noted that the parameters of the associated material properties we used were assumptions. Further *ex vivo* experiments are needed.

The water molecules sprayed by the water jet accelerate drilling into the teeth tissue, which can greatly reduce the generated heat and unpleasant noise. Moreover, this approach avoids iatrogenic infection and reduces the treatment cost compared with that of the traditional turbo high-speed dental drill and water laser drilling. However, the water jet drilling technique, which is economically and environmentally friendly, has just started in the field of dentistry. At present, there are still problems that need to be solved before operating *in vivo*, such as the influence on pulp and oral soft tissue as well as the interference of water mist reflected by the operation on the visual field. Hopefully, in other fields, the water jet drilling technique will become increasingly mature. Colliding water jet is a case in which the water jet drilling technique can drill accurately and safely [44, 45].

The results of this study showed that the water jet drilling with suitable parameters is effective for enamel drilling. It lays a foundation for further research and application of the high-pressure water jet drilling techniques for tooth cavity preparation.
